# Megafaunal variation in the abyssal landscape of the Clarion Clipperton Zone

**DOI:** 10.1016/j.pocean.2018.11.003

**Published:** 2019-01

**Authors:** Erik Simon-Lledó, Brian J. Bett, Veerle A.I. Huvenne, Timm Schoening, Noelie M.A. Benoist, Rachel M. Jeffreys, Jennifer M. Durden, Daniel O.B. Jones

**Affiliations:** a National Oceanography Centre, University of Southampton, Waterfront Campus, European Way, SO14 3ZH Southampton, UK; b Ocean and Earth Science, University of Southampton, National Oceanography Centre, Southampton, UK; c GEOMAR Helmholtz Centre for Ocean Research, D-24148 Kiel, Germany; d School of Environmental Science, University of Liverpool, L69 3GP Liverpool, UK

**Keywords:** Biodiversity, Geomorphology, Polymetallic nodules, Deep-sea mining, Abyssal plains, Sample size, NE Pacific, CCZ, APEI

## Abstract

The potential for imminent polymetallic nodule mining in the Clarion Clipperton Fracture Zone (CCZ) has attracted considerable scientific and public attention. This concern stems from both the extremely large seafloor areas that may be impacted by mining, and the very limited knowledge of the fauna and ecology of this region. The environmental factors regulating seafloor ecology are still very poorly understood. In this study, we focus on megafaunal ecology in the proposed conservation zone ‘Area of Particular Environmental Interest 6′ (study area centred 17°16′N, 122°55′W). We employ bathymetric data to objectively define three landscape types in the area (a level bottom Flat, an elevated Ridge, a depressed Trough; water depth 3950–4250 m) that are characteristic of the wider CCZ. We use direct seabed sampling to characterise the sedimentary environment in each landscape, detecting no statistically significant differences in particle size distributions or organic matter content. Additional seafloor characteristics and data on both the metazoan and xenophyophore components of the megafauna were derived by extensive photographic survey from an autonomous underwater vehicle. Image data revealed that there were statistically significant differences in seafloor cover by nodules and in the occurrence of other hard substrata habitat between landscapes. Statistically significant differences in megafauna standing stock, functional structuring, diversity, and faunal composition were detected between landscapes. The Flat and Ridge areas exhibited a significantly higher standing stock and a distinct assemblage composition compared to the Trough. Geomorphological variations, presumably regulating local bottom water flows and the occurrence of nodule and xenophyophore test substrata, between study areas may be the mechanism driving these assemblage differences. We also used these data to assess the influence of sampling unit size on the estimation of ecological parameters. We discuss these results in the contexts of regional benthic ecology and the appropriate management of potential mining activities in the CCZ and elsewhere in the deep ocean.

## Highlights

• Seafloor geomorphology was important in the structuring of abyssal megafauna.
• Differences in megafaunal community ecology were found between all landscape types.
• Lower megafauna density & diversity in a bathymetric valley than flat and ridge areas.
• Large samples, collected by AUV, were required to make robust ecological conclusions.

## 1 Introduction

The likelihood of polymetallic nodule mining in the Clarion Clipperton Fracture Zone (CCZ) has attracted considerable scientific attention (Levin et al., 2016, Van Dover et al., 2017, Wedding et al., 2015). The potential impacts of mining are likely to extend over extremely large seafloor areas (Aleynik et al., 2017, Glover and Smith, 2003). Such disturbance may lead to major change in the benthic fauna (Jones et al., 2017) and full recovery might take thousands of years (Glasby et al., 1982). Sixteen nodule mining exploration contract areas (75,000 km^2^ each) were granted in the CCZ between 2001 and 2014 by the International Seabed Authority (ISA) (Wedding et al., 2015). The ISA also allocated a series of nine Areas of Particular Environmental Interest (APEIs) beyond these claim areas, where exploitation is prohibited (ISA, 2012). The APEIs were designated to preserve source populations of species for future recolonization of disturbed areas (Lodge et al., 2014). However, the majority of these APEIs remain unstudied; it is not clear if their environmental conditions and faunas are similar to those of the mining claims (Glover et al., 2016a). As a result, improved knowledge of the drivers structuring biological communities in the CCZ is urgently needed to test the presumed functionality and current spatial arrangement of the APEIs system, and to re-assess the regional environmental plan (ISA, 2012).

The CCZ is generally considered as an extensive abyssal plain delimited by the topography of two WSW-ENE trending fracture zones, Clarion and Clipperton. There is a gradual increase in water depth from east (4000 m) to west (5000 m) owing to the sinking of older, cooler oceanic crust to the west (Pushcharovsky, 2006). However, slight variations in spreading rate appear to have shaped the CCZ seafloor into a series of bathymetric highs and lows with a characteristic spacing of 1–10 km, elongated perpendicular to fracture zones (Klitgord and Mammerickx, 1982, Olive et al., 2015). These horst and graben structures shape the CCZ seafloor as a succession of ridges, valleys, and intervening plains. This topographic variation is thought to be generally characteristic of the abyssal environment worldwide (Harris et al., 2014). The very low influx of terrigenous sediments to the CCZ prevents the blanketing of this topography, as may occur on abyssal plains adjacent to continental margins (Smith and Demopoulos, 2003).

Abyssal plains represent some 70% of the world’s seafloor (Harris et al., 2014) and are considered the largest ecosystems on Earth (Ramirez-Llodra et al., 2010). They are poorly explored but appear to have high species richness, including very many undescribed taxa (Smith et al., 2006). Despite their name, abyssal plains can have significant topography that influences the diversity and composition of deep-sea fauna (Durden et al., 2015, Leitner et al., 2017, Stefanoudis et al., 2016). This ecological variation appears to result from the interconnected effects of topographically-driven variation of local current dynamics (Thistle et al., 1991), sediment composition (Durden et al., 2015), and food supply (Smith and Demopoulos, 2003, Morris et al., 2016). However, habitat complexity derived from abyssal landscape geomorphology may have been underappreciated in global estimations of ecological heterogeneity at the deep-sea floor (Durden et al., 2015, Morris et al., 2016); a factor that might be particularly significant to the ecology of the CCZ.

The CCZ appears to have one of the highest levels of deep-sea megafaunal (>1 cm length) species richness (Kamenskaya et al., 2013, Tilot et al., 2018). Morphospecies richness estimations from imagery data can rise above 200 taxa in local assessments (Amon et al., 2016). True species diversity and genetic biodiversity is expected to be much higher (Glover et al., 2015). Given their smaller body size, even higher local diversity is to be expected in the meio- and macrofaunal assemblages of the CCZ (De Smet et al., 2017, Pape et al., 2017). Epifauna, particularly suspension feeders, appear to have higher numerical densities in locations with higher nodule coverage (Vanreusel et al., 2016), with nodule-free areas having an higher proportion of deposit feeders, such as holothurians (Stoyanova, 2012). However, the precise role of nodules, and other local environmental factors, in the ecology of CCZ megafauna is still poorly understood. Faunal composition analyses are scarce, and most quantitative studies have been based on relatively small sampling unit areas (<1000 m^2^) and low replication levels. Meaningful comparison across the CCZ is also hampered by a lack of standardization between studies.

Reliable estimation of ecological parameters relies on appropriate sampling of the populations under investigation. It is often these parameters that serve as the sole basis for conservation management decisions (Andrew and Mapstone, 1987, Magurran, 2004). Investigation of the pros and cons of different sampling strategies is commonplace in terrestrial and shallow-water marine ecology (Andrew and Mapstone, 1987, Buckland et al., 2001, Heck et al., 1975) but rarely tackled in deep-sea studies, except for diversity estimators (Etter and Mullineaux, 2001, Grassle and Maciolek, 1992, Soetaert and Heip, 1990). In part, this lack of research stems from logistic constraints, however, the need is no less. In the CCZ, a key factor may be the very low numerical density of the megafauna, such that identifying an appropriate sampling unit size may be a particular issue (Benoist et al., submitted for publication, Durden et al., 2016a, Durden et al., 2016b). Studies that demonstrate appropriate sampling to support their conclusions are key in ecology, not least those concerned with the regulation of mining activities (Durden et al., 2017a, Levin et al., 2016).

Our study assesses the ecology of the megafauna in the dominant landscape types of APEI6 in the eastern CCZ. We define the landscape types by objective analysis of the bathymetry, establish corresponding sedimentary environmental conditions by direct sampling, and further environmental characteristics and faunal data by extensive seafloor photography from an autonomous underwater vehicle (AUV). In this contribution we examine landscape-type-related variations in standing stock, diversity, and faunal composition and how these parameters, and their interpretation, might vary with the choice of sampling unit size.

## 2 Materials and methods

### 2.1 Study area

The CCZ basin floor is covered by extensive polymetallic nodule fields that add to the seabed heterogeneity and constitute a unique deep-sea habitat (Radziejewska, 2014). Seafloor nodule coverage can be extremely patchy and change drastically over tens of metres (Peukert et al., 2018). Surface sediment is mainly composed of Cenozoic pelagic clays and radiolarian oozes (ISA, 2010). The average carbonate compensation depth (CCD) is around 4500 m (Mewes et al. 2014), although much shallower to the east (∼3500 m) than the west (∼5000 m) (Radziejewska, 2014). Bottom currents are generally weak (<10 cm s^−1^), but direction shifts and periods of stronger flows are not infrequent (Aleynik et al., 2017). The supply of sinking food particles to the seafloor is highly limited as this area is located below some of the most oligotrophic surface waters of the Pacific (Lutz et al., 2007). Food supply to the APEI6 benthos is thought to be higher than in more western CCZ areas (Veillette et al., 2007), yet lower than in more southern areas where spring blooms in surface waters are more pronounced (Lutz et al., 2007, Pennington et al., 2006).

**Fig. 1:**
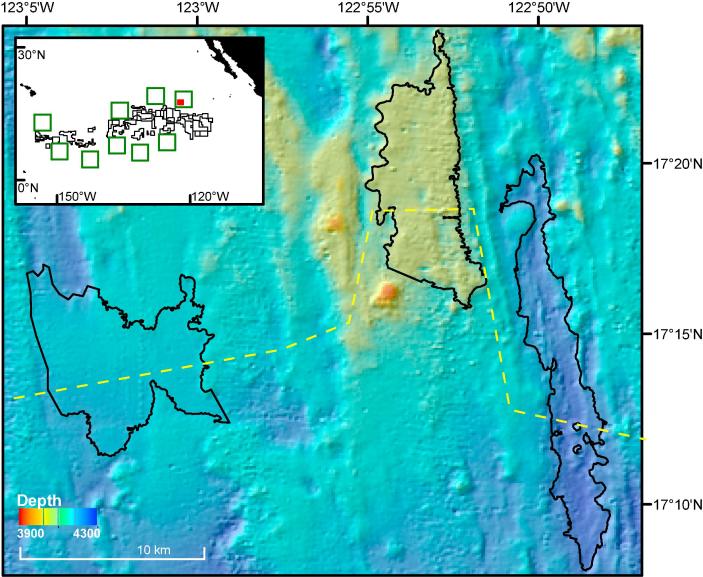
Bathymetric survey chart of the study location within the APEI6 of the CCZ (North Pacific Ocean). Depth (in metres) is indicated by the colour bar. Landscape types mapped using objective classification depicted in dark lines. Yellow dashed line shows seafloor bathymetric profile depicted in Fig. 2. A map of the eastern CCZ is inset, showing exploration licensed areas (black polygons), Areas of Particular Environmental Interest (green polygons), and study location (red square). (For interpretation of the references to colour in this figure legend, the reader is referred to the web version of this article.)

All results reported here relate to the APEI6 area, and were acquired during RRS *James Cook* cruise 120 (Jones, 2015). The survey represented a 5500 km^2^ rectangle of seafloor centred on 17°16′N 122°55′W (Fig. 1), chosen to have similar topographic relief to that in mining exploration contract areas in the central CCZ. Water depth ranged 3950–4250 m, and the seafloor landscape comprised a succession of crenulated ridges and shallow troughs oriented north-south between dispersed level-bottom (<3° slope) areas.

### 2.2 Survey design

#### 2.2.1 Bathymetric mapping and landscape characterisation

**Fig. 2:**
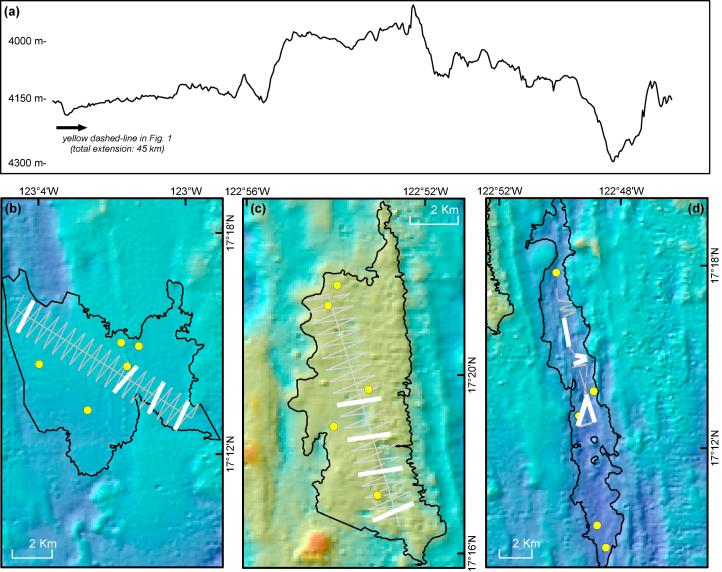
Survey Landscape type study areas investigated at the APEI6. (a) Seafloor bathymetric profile depicted as yellow-dashed line in Fig. 1. No vertical exaggeration was applied. b–d: Detail of sampling operations: grey lines indicate full AUV image survey tracks, thick white lines highlight replicate sampling units selected for analysis, and yellow dots represent coring stations. Study areas surveyed: (b) Flat area. (c) Ridge area. (d) Trough area. (For interpretation of the references to colour in this figure legend, the reader is referred to the web version of this article.)

Multibeam data were collected with the shipboard Simrad EM120 system (191 beams) and processed using CARIS HIPS and SIPS software (TeledyneCARIS; v8.0). The resultant digital elevation model (∼100 m horizontal resolution) was used to calculate a broad bathymetric position Index (bBPI) (Weiss, 2001) and a terrain ruggedness index (TRI) (Wilson et al., 2007) using SAGA v. 2.1.4 software (Conrad et al., 2015). BPI was calculated using an inner radius of 500 m and an outer radius of 10,000 m, and TRI was calculated with a 500 m radius circular neighbourhood. These areas were selected to be representative of the landscape-scale geomorphological variation that was the target of this study. After visual inspection of the resultant datasets, classification thresholds were set to map ridge (bBPI: 50–100; TRI: 0–150), trough (bBPI: −100 to −50; TRI: 0–150), and flat (bBPI: −50 to 50; TRI: 0–50) areas. Contours were drawn using ArcGIS v10 (ESRI, 2011) along the threshold values of each dataset, and used to delimit landscape-type polygons. Three polygons each representing a characteristic landscape type were chosen for stratified-random sampling: Flat area, Ridge area, and Trough area (Fig. 2). Data were projected in Universal Transverse Mercator projection, Zone 10 N, using the World Geodetic System 1984 datum.

#### 2.2.2 Direct sampling

Five sediment sampling stations, with a minimum separation of 100 m, were randomly selected within each study area (Fig. 2b–d). Two Megacore (Gage and Bett, 2005; 10 cm internal diameter) samples were collected per station. Each sample was initially sliced and split by sediment depth. Sediment grain-size distributions were assessed from one core in 0–5 and 5–10 mm depth horizons, by laser diffraction using a Malvern Mastersizer 2000 after homogenisation (grains > 2 mm removed), dispersal in 0.05% (NaPO_3_)_6_ solution, and mechanical agitation. Grain-size distributions obtained for the two horizons were averaged for presentation. The 0–10 mm horizon from the second core were assessed for sediment chemistry. Total carbon (TC) and total nitrogen (TN) contents were measured in duplicate (reproducibility <±5%) using a Carlo Erba NC 2500 CHN Elemental Analyser. Total organic carbon (TOC) was determined after de-carbonation of the samples using the acid HCl vapour method of (Yamamuro and Kayanne, 1995).

#### 2.2.3 Photographic survey

Seafloor photographic images were collected using two digital cameras (FLIR *Grasshopper2*; 2448 × 2048 pixels), one mounted vertically, and one forward oblique facing on the AUV Autosub6000 (Morris et al., 2014). The camera layout and the underwater navigation system were set as described in Morris et al. (2014). The AUV was programmed for a target altitude of 3 m above the seafloor, a speed of 1.2 m s^−1^, and a photographic interval of 850 ms. At the target altitude, individual vertical photographs imaged 1.71 m^2^ of seabed.

We applied a stratified-simple random sampling design (Andrew and Mapstone, 1987) with even sampling effort amongst areas. In each area, a zig-zag survey design (Fig. 2b–d), with random start point, was chosen to maximise sampling efficiency while minimising design-based bias in the spatial distribution of the replicate sampling units (Buckland et al., 2001, Strindberg and Buckland, 2004). A total of 40 sampling units, the straight-line zig and zag sections, were surveyed in each area. Four of these sampling units were then randomly selected in each area for subsequent analysis. Images taken as the vehicle changed course, i.e. junctions between sampling units, were discounted. In the remaining straight-line sections, every second image was discounted to avoid overlap between consecutive images and the risk of double counting. We believe that the various steps of our survey design will have acted to minimise potential spatial autocorrelation, i.e. the double randomisation of sample selection (randomised start position, randomised sampling unit choice) (Strindberg and Buckland, 2004), the two-dimensional coverage provided by the zig-zag design (Foster et al., 2014), and the use of a physically large sampling unit with controlled outer boundaries (Legendre and Fortin, 1989). To ensure consistency in specimen detection, images outside the altitude range 2–4 m were also discounted. The total seabed area analysed from each of the randomly selected sampling units was then standardised to c. 1320 m^2^ (range 1321–1324 m^2^) by random selection from the remaining constituent images, typically 715 photographs (range 555–781; Table A.1). All images used for data generation were colour corrected as described by Morris et al. (2014).

### 2.3 Data analysis

#### 2.3.1 Environmental assessment

Sediment grain size statistics were calculated using Gradistat v.8 software (Blott and Pye, 2001), applying the geometric method of moments (Krumbein, 1936). Mud content was calculated as the proportion of particles < 63 μm. Carbonate content (% sediment dry weight) was calculated from the difference between TC and TOC (assuming all carbonate was CaCO_3_). The ratio of total organic carbon to total nitrogen (C:N) was calculated as the molar ratio.

Nodule seafloor coverage (% cover) and total surface covered by nodules (m^2^) were quantified from AUV imagery using the Compact-Morphology-based poly-metallic Nodule Delineation (CoMoNoD) method (Schoening et al., 2017). CoMoNoD attempts to detect all polymetallic nodules present in an image and calculates their areal extent (cm^2^) based on an ellipsoidal shape projection, to correct for potential underestimation resulting from sediment cover. Only nodules ranging from 0.5 to 60 cm^2^ (i.e. with maximum diameters of ∼1 to ∼10 cm) were considered for analysis to avoid inclusion of large non-nodule formations. Angular-shaped cobbles to large rocks and whale bones (min. diameter > 10 cm) coated in ferromanganese crust were manually counted and measured. Average nodule cover (%) and total nodule area extent (m^2^) were calculated across the selected images of each sampling unit.

#### 2.3.2 Megafauna assessment

Images used for megafauna data generation were reviewed in random order to minimise time or sequence-related bias (Durden et al., 2016a, Durden et al., 2016b). Specimens (>10 mm) were identified to the lowest taxonomic level possible (morphospecies: msp), measured using the BIIGLE 2.0 software (Langenkämper et al., 2017), and assigned to a “nodule-attached” (NA) or “nodule-free-living” (NFL) life habit. To ensure consistency in identification, a megafauna morphospecies catalogue was developed based on an existing CCZ collation (see http://ccfzatlas.com), which was updated and maintained in consultation with international taxonomic experts and by reference to the existing literature (Amon et al., 2017, Dahlgren et al., 2016, Glover et al., 2016b, Kersken et al., 2018). The likely feeding behaviour of each morphospecies was inferred from similar organisms described in the literature (i.e. Iken et al., 2001). Individual metazoan specimen biovolume was estimated, as a proxy for biomass, from two body measurements using the generalised volumetric method described of Benoist et al. (submitted for publication). Despite being comparable in size to metazoan morphospecies, xenophyophores were analysed separately since it is not possible to determine whether they are living from images (Hughes and Gooday, 2004).

A range of ecological parameters were calculated for each replicate sampling unit, including numerical density (ind m^−2^) and proxy biomass density (ml m^−2^ ≈ g fresh wet weight m^−2^). To examine the range of diversity characteristics, Hill’s diversity numbers of order 0, 1, and 2 (Jost, 2006) were calculated as morphospecies richness (S), the exponential form of the Shannon index (exp H́), and the inverse form of Simpson’s index (1/D), using the ‘vegan’ package implemented in R (Oksanen et al., 2018). Additionally, sample-based morphospecies rarefaction curves were fitted using the analytical method proposed by Colwell et al. (2012), using Estimate S v.9.1 software (Colwell, 2013), by randomly resampling without replacement, while exp H́ and 1/D rarefaction curves were calculated with replacement. *K*-dominance curves were also generated to explore dominance patterns (Clarke, 1990).

#### 2.3.3 Statistical analyses

Generalized linear models (GLM) (Dobson and Barnett, 2008) were built to test whether statistically significant variation in environmental or biological parameters was apparent between study areas, using the ‘car’ package (Fox et al., 2016) implemented in R (R Core Team, 2017). Models were fitted with quasi-Poisson errors in non-negative integer metrics (i.e. density, S) with over-dispersion (Gardner et al., 1995), and with normal errors applied to non-integer variables (i.e. mean grain size, exp H́, 1/D) (Freund and Littell, 1981). Differences in proportional metrics (i.e. nodule coverage, mud content, or functional group percentages) were tested with beta-regression models (Ferrari and Cribari-Neto, 2004) using the ‘betareg’ package (Cribari-Neto and Zeileis, 2010). When statistically significant effects were detected in these global test, simultaneous tests were applied to make multiple comparisons between individual study areas, using the ‘multcomp’ package in R (Hothorn et al., 2008). Spearman's rank correlation coefficients were calculated across different biological parameters to investigate potential co-variations between these, using the ‘hmisc’ package (Harrell, 2018). Homogeneity of variance and normality assumptions were verified by visual inspection of model histograms and QQ plots. Statistical significance was reported for p < 0.05.

Variations in community composition between study areas were explored using a range of abundance-based multivariate approaches. The Bray-Curtis dissimilarity measure, based on square-root transformed faunal density, as calculated using the ‘vegan’ package in R, was used throughout these analyses. Non-metric multidimensional scaling (nMDS) ordination was used to visualise variations (‘vegan’ package in R). A one-way permutational MANOVA (PERMANOVA) analysis (Anderson, 2001), with follow-up pair-wise tests, was used to test for statistically significant variations in assemblage composition between study areas, using PRIMER v.7 (Clarke and Gorley, 2015). A SIMPER (‘‘similarity percentages’’) analysis was performed to assess morphospecies contribution to between-group dissimilarity (‘vegan’ package in R).

#### 2.3.4 Megafauna sampling effort evaluation

To assess the reliability of the biological survey developed in the present study, we investigated the effect of varying sampling unit size (seabed area or individuals covered per sample unit) on the accuracy (i.e. stabilization of mean value) and precision (i.e. coefficient of variation: CV) of different ecological parameters. Image data were first pooled within study area (i.e. across sampling units) and then randomly resampled 1000 times with or without replacement (depending on the target parameter and approach used: see below) into new sampling unit sets of increasing image number size. The mean (or median), the precision (CV), and the confidence intervals (95%) of each parameter were calculated at each sample unit size, together with the mean total seabed area and individuals represented by the images composing each subset.

Morphospecies rarefaction curves were fitted using the analytical method proposed by Colwell et al. (2012), using Estimate S v.9.1 software (Colwell, 2013), by randomly resampling image sets of increasing size without replacement. Rarefaction curves were interpolated and extrapolated up to 3000 individuals sampled, to balance for differences in fauna densities. Additionally, curves were extrapolated up to 15,000 m^2^ per study area (see Fig. A.2). The autosimilarity approach proposed by Schneck and Melo (2010), as implemented in the seabed image case by Durden et al. (2016a), was applied to evaluate precision in assemblage description. At each sampling unit size, Bray-Curtis dissimilarity was computed between two groups of images, each randomly selected without replacement. Metazoan density, biomass density, and exp H́ and 1/D indexes were computed by bootstrapping image subsets (Buckland et al., 2001). Custom R scripts and the ‘vegan’ package were used to process image data and calculate all ecological indices.

## 3 Results

### 3.1 Environmental assessment

Surface sediments (0–10 mm horizon) were dominated by radiolarian-bearing pelagic clay to fine silt particles (diameter < 7.8 μm; 58–68% of particles), and medium to very coarse silt grains (diameter 7.8–63 μm; 28–39% of particles). Mean and median particle size, and mud proportion showed no statistically significant variation between areas, though larger value ranges were evident among the Ridge area samples (Table 1). Subsurface sediments (>50 mm horizon) in the Ridge and Trough showed much greater variability in grain size distributions than those in the Flat area (Fig. A.1; Table A.2). Relative proportions of TOC, TN, and CaCO_3_ were almost homogenous across the study areas; no statistically significant differences were detected between study areas (Table 1).

**Table 1:** Environmental and biological features assessed for each APEI6 landscape type, with detail on the general linear models (GLM) applied to explore variations of these parameters between study areas. **Sediment parameters:** measured from surface sediment (0–10 mm) and shown as: mean (minimum - maximum) obtained amongst all replicate Megacore samples (n = 5) collected in each area. *Parameters*: particle size; mud content (particles < 63 μm) percentage; percentages of total organic carbon (TOC) and CaCO_3_; and molar C_org_/Total nitrogen ratio. **Image parameters:** measured from seafloor imagery data and shown as: mean (95% confidence intervals: lower – upper) calculated amongst all replicate image samples (n = 4) collected in each area. *Parameters*: seafloor percentage cover and total nodule area calculated using the CoMoNoD algorithm on seabed imagery (see text); density of non-nodule (>10 cm) hard substrata (boulders and whale bones); total density and proportion of metazoan and xenophyophore individuals (>10 mm) split in different functional (SF: suspension feeders; DF: deposit feeders) and life-habit (NA: nodule-attached) categories; biomass (grams of fresh wet weight) density inferred using the generalised volumetric method (see text); and diversity: richness, exponential Shannon (exp H′), and inverse Simpson (1/D) indices. Error fit types: normal (G), beta (B), quasi-Poisson (QP). Significance level: p < 0.05 (*), p < 0.01 (**).

	Flat	Ridge	Trough	Error fit	F-value
Sample parameters					**(*F_2,14_*)**
Sediment mean grain size (μm)	8.1 (7.7–8.2)	9.5 (6.8–17.6)	9.2 (8–12.2)	G	0.34
Sediment mud content (%)	92.6 (91.7–93.8)	92.5 (79.9–95.7)	90.7 (85.6–93.2)	B	1.01
Sediment TOC (%)	0.42 (0.39–0.44)	0.41 (0.35–0.45)	0.44 (0.39–0.49)	B	0.8
Sediment C_org_ TN^−1^	4.0 (3.8–4.3)	3.8 (3.6–4.0)	4.1 (3.7–4.5)	B	0.85
Sediment CaCO_3_ (%)	0.33 (0.24–0.53)	0.48 (0.26–0.66)	0.36 (0.26–0.48)	B	0.5

Image parameters					**(*F_2,11_*)**
Nodule surface size (cm^2^)	2.6 (2.3–2.9)	2.0 (1.7–2.3)	2.1 (1.6–2.6)	G	2.57
Nodule seabed cover (%)	10.1 (7.2–12.3)	6.3 (4.3–8.6)	3.8 (1.9–6.5)	B	6.73**
Nodule seabed cover (m^2^)	133.8 (95.4–162.6)	83.0 (56.4–113.8)	50.1 (24.5–86.4)	G	4.82*
Other hard substrata (items ha^−1^)	62 (28–102)	682 (230–1132)	64 (30–102)	QP	10.26**
Metazoan density (ind m^−2^)	0.49 (0.42–0.54)	0.47 (0.41–0.53)	0.32 (0.25–0.39)	QP	5.23*
Metazoan biomass (g fwwt m^−2^)	1.6 (1.1–2.1)	2.9 (1.5–4.2)	2.1 (1.0–3.2)	G	0.79
Metazoan richness (S)	70.5 (67.2–74.0)	64.8 (61.0–68.5)	59.5 (50.5–68.5)	QP	2.09
Metazoan exp H′	29.7 (27.0–32.3)	28.3 (25.5–31.5)	23.4 (18.3–28.4)	G	2.33
Metazoan 1/D	16.4 (14.2–18.5)	16.4 (13.2–19.6)	9.7 (6.2–13.2)	G	4.66*
Metazoan NA (ind m^−2^)	0.34 (0.29–0.38)	0.28 (0.23–0.35)	0.19 (0.13–0.25)	QP	5.33*
Metazoan NA (%)	69.3 (60.9–74.4)	60.0 (50.2–67.3)	57.2 (48.2–65.5)	B	2.49
Metazoan SF density (ind m^−2^)	0.39 (0.34–0.44)	0.34 (0.29–0.39)	0.25 (0.19–0.31)	QP	4.25*
Metazoan SF (%)	79.8 (77.9–81.6)	73.6 (69.6–76.1)	77.2 (74.8–79.5)	B	5.33*
Metazoan DF density (ind m^−2^)	0.07 (0.07–0.08)	0.10 (0.09–0.11)	0.05 (0.04–0.07)	QP	13.90**
Metazoan DF (%)	15.9 (14.4–17.4)	21.6 (18.5–24.8)	17.2 (14.9–19.4)	B	5.56*
Xenophyophore density (ind m^−2^)	2.22 (1.54–2.99)	4.09 (3.55–4.60)	1.33 (0.48–2.6)	QP	5.94**
Xenophyophore NA (ind m^−2^)	1.15 (0.75–1.64)	1.36 (1.01–1.71)	0.52 (0.15–1.14)	QP	2.22
Xenophyophore NA (%)	50.7 (47.5–54.2)	32.8 (28.3–37.2)	32.7 (24.3–41.3)	B	10.22**

The polymetallic nodules observed during the present study were of an ellipsoidal-flat shape with smooth surfaces. Mean nodule surface area was 2.5 cm^2^, with most nodules < 5 cm^2^ (90%), and very few > 10 cm^2^ (1%). Nodules in the Flat were larger than in the other areas, though not significantly so (Table 1). Average nodule cover was 6.4% and ranged from nodule-free to 37%. The highest mean nodule coverage was recorded in the Flat area (Table 1), although both the within-sampling unit and within-area deviations for this metric were high (Table A.1). Nodule coverage did exhibit a statistically significantly difference between study areas (Table 1), with a statistically significant pair-wise difference between the Flat and Trough areas (Tukey, p < 0.05). Larger (>60 cm^2^ in surface) hard substratum formations coated in ferromanganese crust were especially common in the Ridge area, where angular cobbles, boulders, and whale bones were about ten times more abundant than in the other study areas (Table 1). However, the inclusion of these structures (total survey area surface <10 m^2^) to the total hard-substratum availability of each sample unit was negligible, even in Ridge samples.

### 3.2 Megafauna assessment

#### 3.2.1 Metazoan fauna

**Fig 3:**
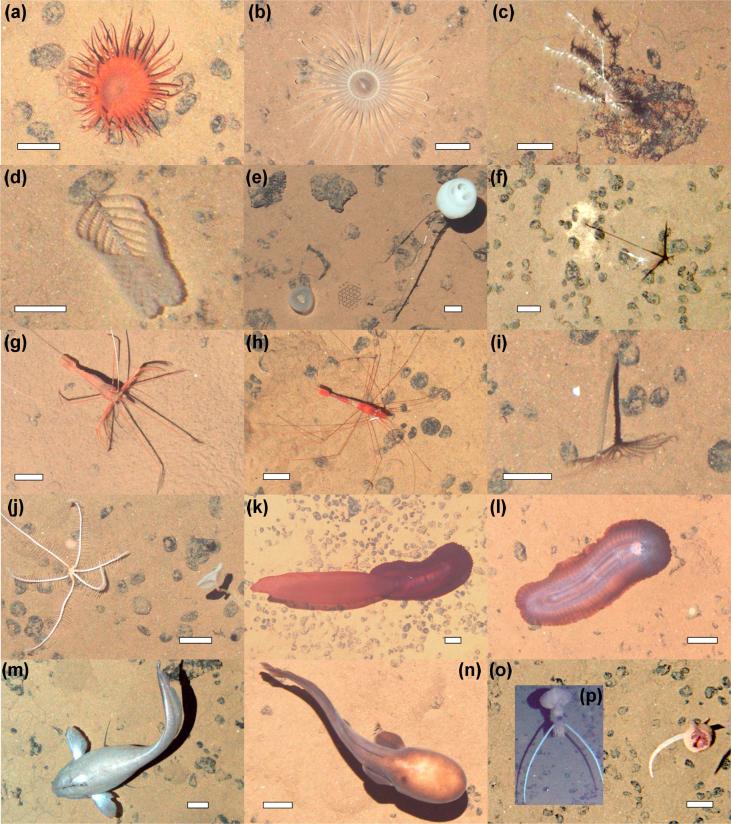
Examples of metazoan megafauna photographed at the APEI6 seafloor during AUV survey. Scale bars representing 50 mm. (a) Actiniaria msp-6. (b) Actiniaria msp-13. (c) *Bathygorgia* cf. *profunda*. (d) *Abyssopathes* cf. *lyra.* (e) Left: *Chonelasma* sp.; right: *Hyalonema sp*. (f) *Cladorhiza* cf. *kensmithi*. (g) *Bathystylodactylus* cf. *echinus*. (h) *Nematocarcinus* sp. (i) Sabellida msp-1 (polychaete). (j) Left: *Freyastera* sp.; right: *Caulophacus* sp. (k) *Psychropotes* cf. *longicauda.* (l) *Benthodytes* cf. *typica*. (m) *Coryphaenoides* sp. (n) *Typhlonus nasus*. o and p: probable new *Mastigoteuthis* sp. Same specimen photographed with different cameras: (o) vertical view; (p) oblique view (Image taken ∼1″ prior to the vertical shot).

A total of 6740 megafauna individuals (>10 mm) were recorded in the 15,840 m^2^ of seabed examined during the present study (Table 2). Megafauna were classified into 129 morphospecies and 11 higher taxonomic categories (i.e. Order, Family; Table 2). Rare taxa (≤3 records) represented a third of the total morphospecies richness. The fauna observed (Fig. 3) were predominantly cnidarians (25 msp; 0.18 ind m^−2^, ∼70% of which were Alcyonacea bamboo corals), sponges (27 msp; 0.07 ind m^−2^), annelids (9 msp; 0.04 ind m^−2^), bryozoans (4 msp; 0.04 ind m^−2^), and echinoderms (32 msp; 0.04 ind m^−2^). Mollusc, crustacean, fish, tunicate, and ctenophore morphospecies were also recorded at lower densities (<0.03 ind m^−2^; Table 2). The metazoan fauna was primarily composed of suspension feeders (78%) and deposit feeders (16%), while predators and scavengers were scarce (4%). Almost 80% of suspension feeding individuals were found attached to polymetallic nodules or other hard substrata. The proportion of nodule-attached individuals was > 70% of the total abundance in 37 morphospecies. These “nodule-dwelling” taxa constituted 70% of the total abundance, and 30% of the total richness recorded.

**Table 2:** Total abundance and taxonomical classification of metazoan morphospecies groups sampled at each APEI6 study area. Abundances are split per life habit: attached to hard-substrata (NA); nodule-free living (NFL). (*) Note that “Group” level taxonomical classification is not hierarchical; ranges from Class to Family level, to simplify tabulation.

Phylum/Class	Group	Morphospecies	Flat		Ridge		Trough	
	(*)	(n)	NFL	NA	NFL	NA	NFL	NA
Ctenophora	Tentaculata	2	1		1			
Porifera	Porifera	10	26	45	33	40	52	35
	Demospongiae	7	42	126	53	119	174	342
	Hexactinellida	9	8	19	19	4	17	9
Cnidaria	Scyphozoa	2	5				6	
	Actiniaria	14	49	310	39	249	37	98
	Alcyonacea	6	107	821	125	633	52	252
	Antipatharia	1		1		1		
	Ceriantharia	2	8	3	2	1	5	1
	Pennatulacea	1	2	1	1		1	
Bryozoa	Cheilostomatida	4	19	251	44	226	25	95
Annelida	Echiura	3	21		20		10	
	Polychaeta	5	63	152	60	173	34	104
Mollusca	Bivalvia	1	74		140		66	
	Gastropoda	2	8		1		3	
	Octopoda	1			1		1	
	Scaphopoda	1	19		7		8	
	Teuthida	1	29		29		22	
Arthropoda	Crustacea	–	33		36		38	
	Amphipoda	3	12		11		11	
	Cirripedia	2	2	23	2	14	3	7
	Copepoda	2	12		2		8	
	Decapoda	8	43		20		30	
	Isopoda	1	16		17		14	
	Mysida	1	7		8		3	
Echinodermata	Asteroidea	5	14		4		4	
	Crinoidea	6	1	12	4	20	5	19
	Echinoidea	5	60		79		45	
	Holothuroidea	11	32		19		16	
	Ophiuroidea	4	78		161		38	
Chordata	Tunicata	2	3	6	1	1	3	7
	Actinopterygii	7	23		18		15	

TOTAL		129	817	1770	957	1481	746	969

##### 3.2.1.1 Patterns in faunal distribution

Mean metazoan density exhibited a statistically significantly difference between study areas (Table 1), with densities in Flat and Ridge areas higher than those in the Trough (Tukey, p < 0.05). We detected statistically significantly higher densities of suspension feeders in the Flat area compared to the Trough, and statistically significantly higher densities of deposit feeders in the Ridge than in the other study areas (Tukey, p < 0.05). Mean density and proportion of predators and scavengers was similar in all study areas (Table 1). Although the proportion of the fauna attached to nodules was not statistically significantly different between study areas (Table 1), the densities of nodule-attached individuals were statistically significantly higher in the Flat than in the Trough (Tukey, p < 0.01). The mean biomass density recorded across all sampling units was 1.22 g fwwt m^−2^ (in c. 1320 m^2^ observed), with no statistically significant difference detected between study areas (Table 1).

**Fig. 4:**
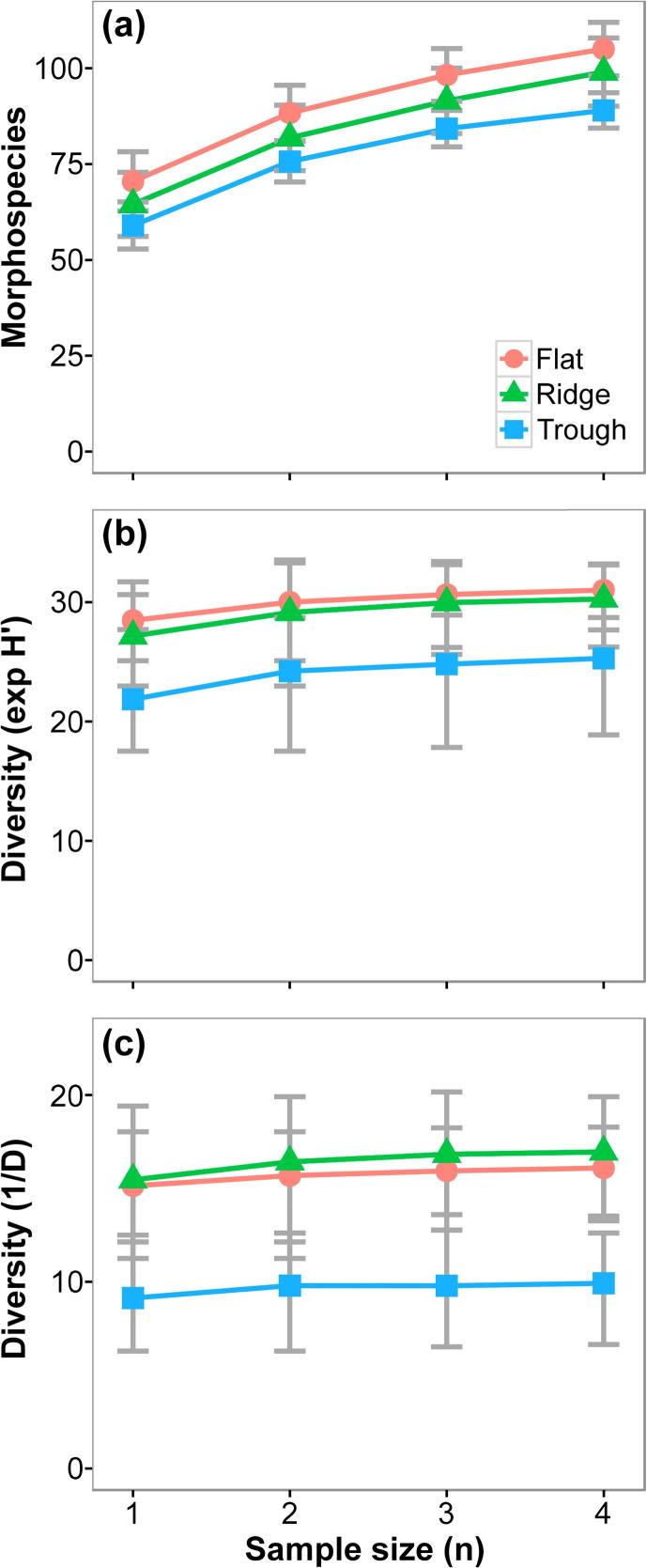
Sample-based diversity accumulation curves calculated for each APEI6 study area. Fauna occurrences of each replicate sample were randomly resampled (with or without replacement) 1000 times at each sampling effort level (n = 1–4). (a) Species rarefaction calculated without replacement. (b) Exponential Shannon index, calculated with replacement. (c) Inverse Simpson index, calculated without replacement. Error bars represent 95% confidence intervals between runs.

**Fig. 5:**
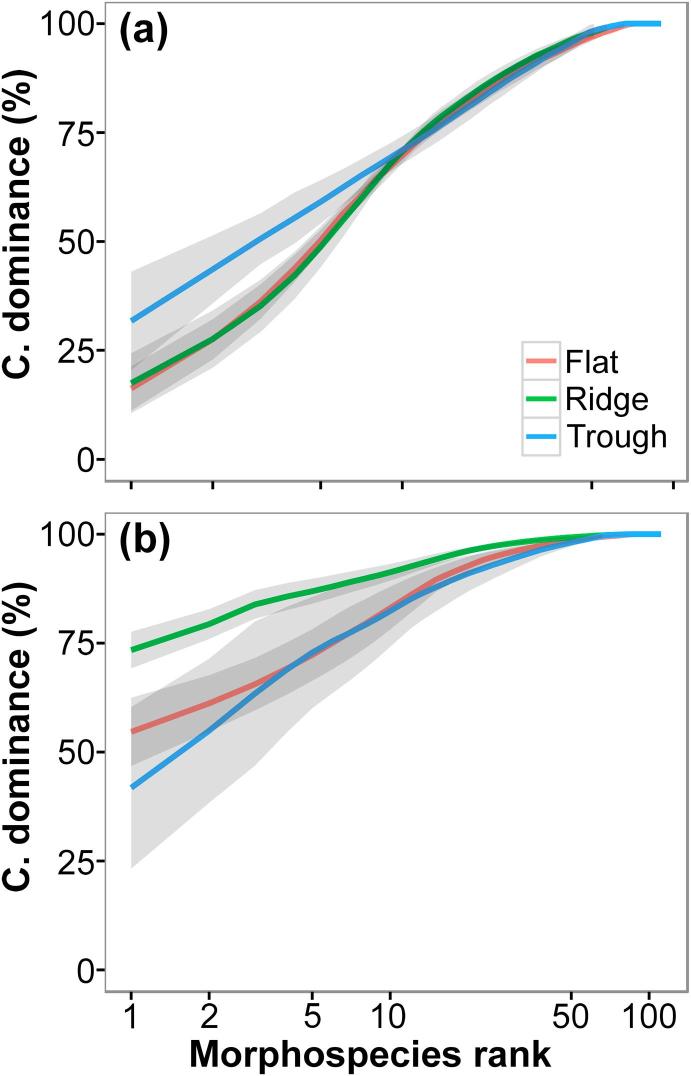
Morphospecies *K*-dominance curves calculated for each APEI6 study area. Curve lines represent cumulative rank abundances calculated as the mean amongst the four replicate samples analysed for each area. Shadowing represents 95% confidence intervals. (a) Curves calculated including only metazoan fauna. (b) Curves calculated including metazoans and xenophyophores.

Mean morphospecies richness (S) was higher in the Flat, though we found no statistically significant difference between study areas (Table 1). Sample-based morphospecies rarefaction curves showed that this pattern was consistent at whole study level (Fig. 4a), and extrapolation of these curves predicted the same scenario even when triplicating the total sampling performed per study area (Fig. A.2). Variations in diversity between study areas were more evident at progressively higher Hill’s numbers (q > 0). Mean exp H′ and 1/D indices were higher in the Flat and the Ridge areas compared to the Trough, although these differences were statistically significant only for the 1/D index (Table 1). These patterns were consistent at whole study level (Fig. 4b–c). We also detected greater morphospecies dominance in the Trough area, and relatively more even taxa abundances in the Flat and Ridge areas (Fig. 5a).

##### 3.2.1.2 Variations in community composition

**Fig. 6:**
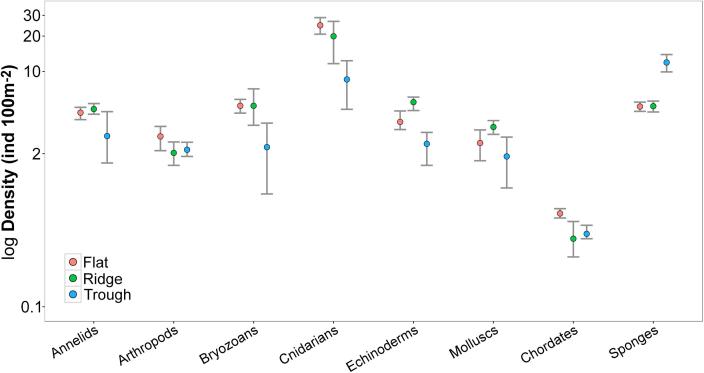
Density variations of different metazoan taxonomic groups between APEI6 study areas. Points represent the mean density of each group calculated amongst the four replicate samples analysed for each area. Error bars represent 95% confidence intervals.

**Fig. 7:**
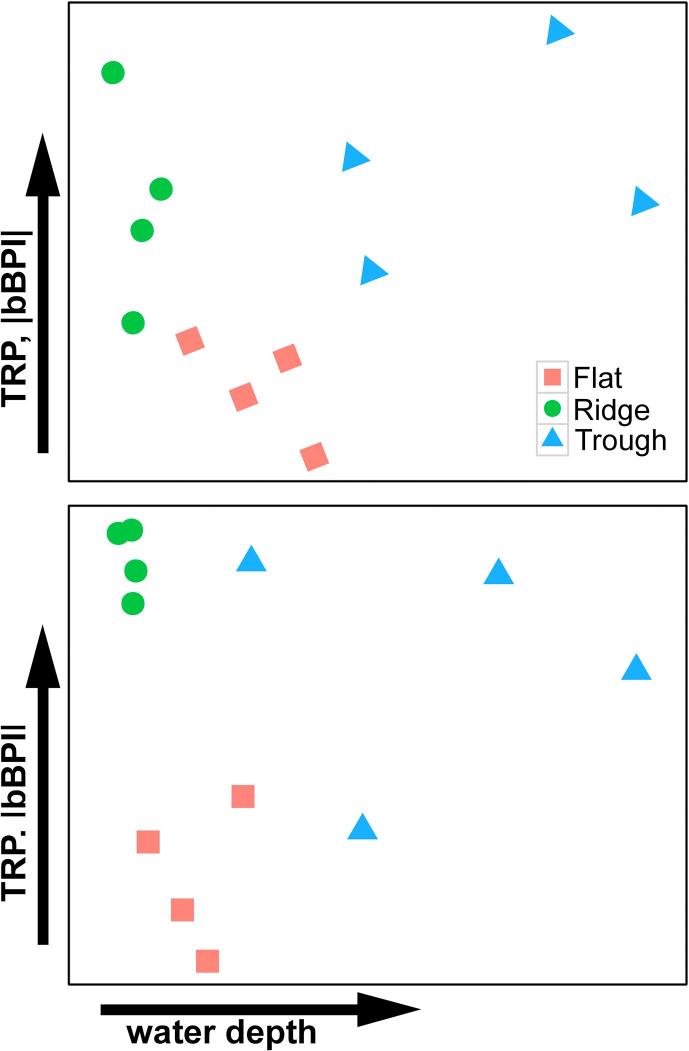
Interpreted megafauna morphospecies composition nMDS for APEI6 samples. Two-dimensional representations of nMDS developed on Bray-Curtis resemblance matrix calculated from square-root transformed megafauna composition by abundance data. (a) nMDS plot developed including only metazoan fauna. (b) nMDS plot developed including metazoans and xenophyophores. Arrows indicating the (non-linear) trend in water depth and bathymetric derivatives suggested for each axis.

Cnidarians, sponges, bryozoans, and echinoderms showed the clearest variations in density between study areas (Fig. 6). In total, 54% of the morphospecies recorded were present in all three study areas, 22% were noted in only two areas, and 24% were detected in only one area. Most (70%) of the single area records were singletons (Fig. A.3) and the rest rare morphospecies (≤5 occurrences). Nevertheless, a statistically significant difference in faunal composition was detected between the study areas (PERMANOVA, R^2^ = 0.39, *p* < 0.001) (Fig. 7a), with statistically significant differences apparent in paired comparisons between the Trough and the other study areas (pair-wise PERMANOVA, R^2^ = 0.36–0.37, p < 0.05). SIMPER analysis showed that variations in the density of the most dominant 10–15 morphospecies were consistently responsible for 70% of the dissimilarity between study areas, but three morphospecies in particular, a sponge (Porifera msp-5) and two soft corals (*Lepidisis* msp and *Callozostron* cf. *bayeri*), contributed most to the dissimilarity. Total density of Porifera msp-5 in the Trough (8.7 ind 100 m^−2^) was four times higher than in the Ridge and Flat areas; total density of *Lepidisis* msp in the Flat (3.8 ind 100 m^−2^) was four times higher than in the Ridge and 20 times higher than in the Trough areas; while total density of *C*. cf. *bayeri* in the Ridge and the Flat (∼2.5 ind 100 m^−2^) was four times higher than in the Trough area.

##### 3.2.1.3 Sampling unit size evaluation

**Fig. 8:**
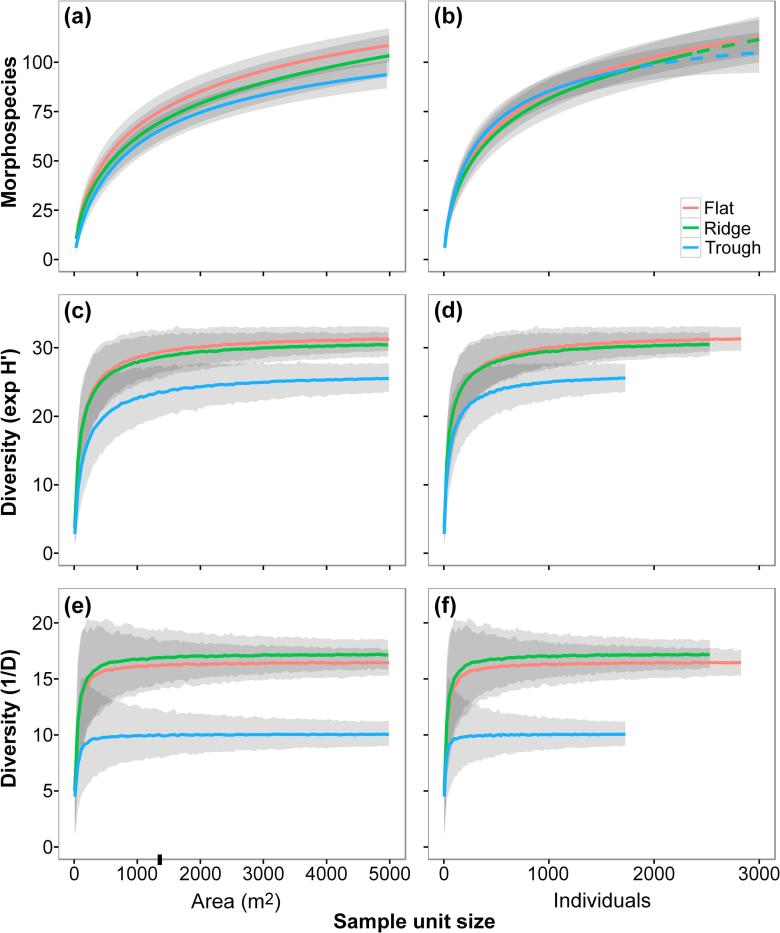
Variation of the different metazoan community diversity indices used in the present study, as a function of the seabed area or number of individuals encompassed by the sample unit size. Lines represent mean values across the 1000 randomisations performed at each sample unit size increase, for each study area collated sample (n = 3) (see methods). Shadowing representing 95% confidence intervals. Ticks on x-axis indicate the sampling unit size used in the present study (replicate sample area = 1320 m^2^). **a and b**: Rarefied metazoan morphospecies accumulation curves. (a) Area-based accumulation curves. (b) Individual-based accumulation curves. Dashed lines represent sample extrapolation. **c and d**: Variation of metazoan exp H′ diversity index. (c) Area-based mean exp H′. (d) Individual-based mean exp H′. **e and f**: Variation of metazoan 1/D diversity index. (e) Area-based mean 1/D. (f) Individual-based mean 1/D.

**Fig. 9:**
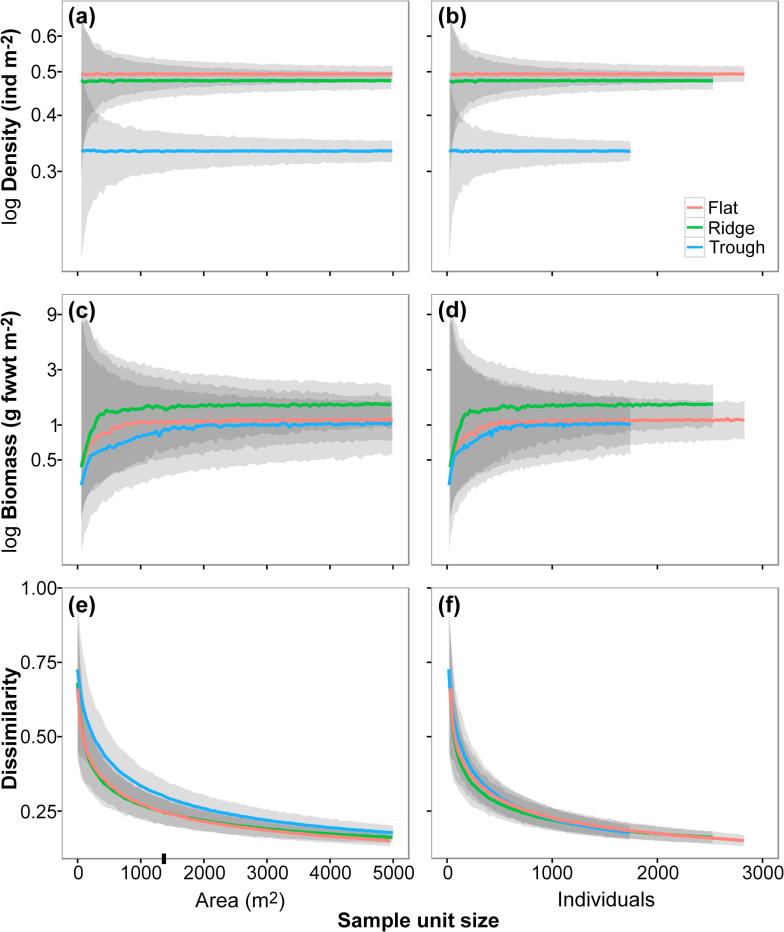
Variation of the different metazoan community parameters used in the present study as a function of the seabed area or number of individuals encompassed by the sample unit size. Lines represent mean or median values across the 1000 randomisations performed at each sample unit size increase, for each study area collated sample (n = 3) (see methods). Shadowing representing 95% confidence intervals. Ticks on x-axis indicate the sampling unit size used in the present study (replicate sample area = 1320 m^2^). **a and b**: Variation of mean metazoan density. (a) Area-based mean density. (b) Individual-based mean density. **c and d**: Variation of median metazoan biomass concentration. (c) Area-based median biomasss. (d) Individual-based median biomass. **e and f**: Autosimilarity curves showing mean Bray-Curtis dissimilarity index calculated amongst pairs of metazoan samples. (e) Area-based autosimilarity curves. (f) Individual-based autosimilarity curves.

Estimates of most of the ecological parameters assessed were stable at the sampling unit size used in the present study (c. 1320 m^2^ of seabed) (Fig. 8, Fig. 9). The maximum precision (CV) reached by each parameter with increasing sampling unit size ranged from 0.02 to 0.30 (Fig. A.4); increases in precision were modest for most parameters with sampling unit sizes > 300 individuals (700–900 m^2^), except for autosimilarity, which required smaller sizes (>150 individuals; 300–450 m^2^) to reach a stable precision (Fig. A.5). Analysis of accuracy yield more variable results. Estimation of mean taxa richness required the largest unit size to stabilise (>500 individuals; 1000–1500 m^2^) (Fig. 8a-b), while faunal density required the smallest (>30 individuals; 50–100 m^2^) (Fig. 9a-b). Mean autosimilarity required unit sizes > 500 individuals (1000–1500 m^2^) to stabilise (Fig. 9c–f); at that size autosimilarity was >70%. Accuracy of biomass density estimates differed between study areas: sampling unit sizes >500 individuals were required for stabilisation of median values in the Flat and Trough samples, while stabilisation in the Ridge occurred >250 individuals. Mean exp H′ stabilized with unit sizes >350 individuals (700–1000 m^2^) (Fig. 8c and d), while mean 1/D stabilised with >200 individuals (400–600 m^2^) (e–f).

#### 3.2.2 Xenophyophore fauna

**Fig. 10:**
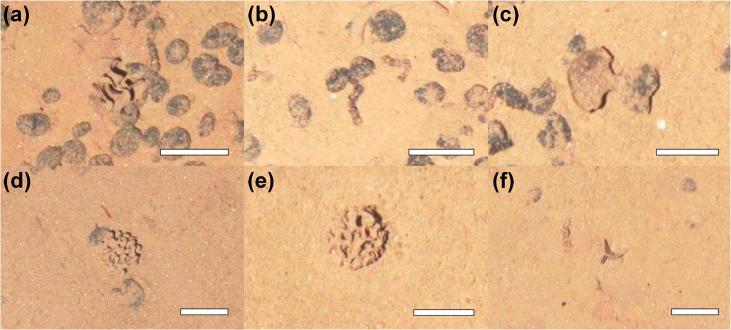
Examples of xenophyophore megafauna photographed at the APEI6 seafloor during AUV survey. Scale bars representing 50 mm. (a) *Reticulammina* msp. (b) *Aschemonella monile.* (c) Fan-shaped *Psammina* msp. (d) Indeterminate Psamminid msp, possibly *Shinkaiya* or *Syringammina.* (e) *Syringammina* cf. *limosa.* (f) Triradiate *Psammina* msp, possibly *P. multiloculata*.

Xenophyophore tests (Fig. 10) numerically dominated the megafauna recorded during the present study; being overall, six times more abundant than metazoans, and reaching a peak density of 17 ind m^−2^ in an image from the Ridge area. Mean xenophyophore density exhibited a statistically significantly difference between study areas (Table 1), with densities in the Ridge higher than those in the Trough (Tukey, p < 0.01). The recently described species *Aschemonella monile* (Gooday et al., 2018) (Fig. 10b) dominated the fauna, having mean densities of 3.27, 1.51, and 0.85 ind m^−2^ in the Ridge, Flat, and Trough areas respectively. The numerical dominance of xenophyophores has substantial impact on the perception of relative faunal diversity among the study areas (Fig. 5b), inclusion of these foram taxa markedly increased rank 1 dominance (Berger-Parker index) in the Flat and Ridge areas, indicating a very substantial reduction in diversity in the Ridge area particularly.

Xenophyophores were classified in 23 morphospecies. Xenophyophore faunal composition exhibited statistically significant variation between study areas (PERMANOVA, R^2^ = 0.55, p < 0.001), with statistically significant differences detected in all paired comparisons (pairwise PERMANOVA, R^2^ = 0.39–0.61, p < 0.05). Joint analysis of xenophyophore and metazoan faunal composition yielded comparable results (Fig. 7b) to those obtained from the analysis of metazoan taxa only (Fig. 7a); statistically significant variations between study areas (PERMANOVA, R^2^ = 0.48, p < 0.001) were led by differences between the Trough and the other study areas (pairwise PERMANOVA, R^2^ = 0.37–0.45, p < 0.01).

## 4 Discussion

### 4.1 Environmental setting at the APEI6

The high homogeneity in particle size and nutrient availability found across the APEI6 study areas suggests that these factors may be consistent over scales broader than the tens of kilometres between areas studied here. Our results were somewhat unexpected since variations in sediment grain-size distributions and particulate organic matter have been reported between landscape types in previous assessments in the north Atlantic abyss (Durden et al., 2015, Morris et al., 2016), where bottom current speed ranges (Vangriesheim et al., 2001) are comparable to those expected at the APEI6, but sediments were coarser and more heterogeneous. Surface sediment particle sizes at the APEI6 were comparable in range to those found in eastern CCZ contract areas (Khripounoff et al., 2006, Mewes et al., 2014, Pape et al., 2017). Although sediments in these more southerly areas exhibit bimodal particle size distributions, being primarily composed of clays and fine silts (<6.3 μm), but with higher proportions of sands (>63 μm) than at the APEI6. Ranges of TOC (0.41–0.44%) and C:N ratios (3.8–4.1) were also comparable to those reported in eastern CCZ contract areas (Khripounoff et al., 2006, Mewes et al., 2014, Pape et al., 2017). This suggests that the sedimentary environment of the APEI6 may be generally representative of the environment found at a larger scale (i.e. eastern CCZ), although further work in other contract areas would be required to draw more precise conclusions in this regard.

Variations in nodule abundance could be indicative of environmental change between study areas. Locally stronger bottom-water currents reducing deposition rates are presumed to enhance nodule formation (Mewes et al., 2014, Skornyakova and Murdmaa, 1992). Higher nodule abundances on modest slopes and elevated seafloors, such as the Flat and the Ridge areas, have commonly been linked with low sedimentation rates (Frazer and Fisk, 1981, Mewes et al., 2014). Yet convergent channelling of bottom currents in bathymetric valleys, such as the Trough area, has also been suggested to limit deposition enhancing nodule growth (Peukert et al., 2018). The more irregular nodule coverage we observed in the Ridge ( Table A.1) concurs with previous descriptions of hilltop environments at the CCZ (Jung et al., 2001, Margolis and Burns, 1976, Skornyakova and Murdmaa, 1992). In these, current circulation over rugged seafloor can generate scattered redistribution of surface materials (Jung et al., 2001, Nasr-Azadani and Meiburg, 2014, Peukert et al., 2018), which may have reduced the sediment blanketing of hard substrata (i.e. rock fragments, whale bones) and trace fossils (Durden et al., 2017b) within the Ridge.

### 4.2 Sampling unit size evaluation

Improved precision with increasing sampling unit size was apparent in all parameters (Fig. A.5), as was expected from previous image-based assessments (Durden et al., 2016b), but the accuracy of each parameter (Fig. 8, Fig. 9) showed a different sensitivity to this factor. The sampling unit size we used in this study (c. 1320 m^2^ of seafloor) appeared to be sufficiently large for reliable estimation of faunal density, diversity of higher orders (exp H′, 1/D), and community dissimilarity, but was potential below ideal for the assessment of taxon richness and biomass density, i.e. not all samples contained the ≥500 individuals suggested by our analysis for these parameters (Table A.1). The need for larger sampling unit sizes in the estimation of taxon richness and biomass density is a relative rarity effect. The comparatively high taxon richness that we note in APEI6, draws the tail of the species abundance distribution far out to the right, a common observation in abyssal studies (Smith and Demopoulos, 2003). Similarly, the rarity of the very largest organisms, the far right tail of the body size distribution, has substantial impact on biomass density estimates (e.g. Bett, in press). Despite their relative rarity, these large megafaunal species play an important ecological role in these deep-sea environments (Billett et al., 2001, Ruhl et al., 2008).

Our results underline that sampling unit size evaluation is important for assessing the reliability and comparability of ecological patterns inferred in environments where faunal density is low. Minimum sampling unit sizes for appropriate parameter estimation were highly variable (30–500 individuals; 100–1500 m^2^ per sample unit) in the present study, driven by the character of each parameter (see also Durden et al., 2016a, Durden et al., 2016b). Consequently, considerable care must be taken when working with data from physically small sampling units, and particularly when making comparisons between studies employed very different sampling unit sizes. There is a clear need for the appropriate tuning of the sampling unit size in abyssal ecology, especially at the CCZ, where the resultant data may have a substantial influence on conservation policy (Durden et al., 2017a, Levin et al., 2016). To date, little attention has been given to this topic in the CCZ (Stoyanova, 2012, Tilot et al., 2018, Vanreusel et al., 2016, Wang and Lu, 2002), this will undoubtedly complicate attempts to synthesise data across the region (Amon et al., 2016). For example, megafauna assessments performed by Tilot et al., 2018, Stoyanova, 2012 reported densities an order of magnitude lower than those of Vanreusel et al. (2016) for the same areas. The application of improved imaging systems may have increased the apparent megafauna densities, and influenced corresponding diversity estimations. These points stress the need for a standardization of both assessment method and morphotype taxonomy across the CCZ, to enable more reliable comparisons between the various APEI and claim areas and simplify the detection of possible biogeographic boundaries in the CCZ.

### 4.3 Landscape ecology of metazoan megabenthos

Differences in megafauna density across the landscape types studied were predominately driven by variations in suspension feeder abundance (Table 1), particularly sessile cnidarians (Fig. 6). Potential topographically-enhanced bottom water current speeds have previously been suggested to promote the development of suspension feeding fauna in the abyss (Durden et al., 2015, Smith and Demopoulos, 2003, Thistle et al., 1985). Suspension feeders usually dominate the megabenthos in the CCZ and show higher abundances in areas with higher nodule density (Amon et al., 2016, Stoyanova, 2012, Vanreusel et al., 2016). Factors promoting higher nodule densities also enhance the development of suspension feeders (Vanreusel et al., 2016); for example, in the present study most suspension feeders (80%) were attached to nodules. Suspension feeder density, and relative abundance, may therefore be related to both the availability of hard substrata and local enhancements in bottom water currents, and that the latter two factors may themselves be related. These factors suggest that low slopes or elevated topographies, as found at the Flat and Ridge areas, enhance suspension feeder densities increasing the overall metazoan standing stock of these areas, as compared to depressions, like the Trough area.

Variations in functional composition between study areas were driven by the distribution of deposit feeder fauna, suggesting enhanced resource availability for this group in the Ridge. This could indicate a higher food supply at the more elevated seafloor of the Ridge, owing to less particulate organic carbon loss during sinking (Smith et al., 2008a), but this is likely to be a small effect at abyssal depths for changes of few hundred meters (Lutz et al., 2007). Moreover, sediment TOC exhibited no statistically difference between study areas, nor was there a statistically significant difference in the C:N ratio. This suggests that, if there were variations in food supply for deposit feeders, these may either have occurred at a finer spatial scale (i.e. patch accumulations: Lampitt, 1985, Smith et al., 1996), or be related with the quality rather than the quantity of the available resource (Ginger et al., 2001).

Deposit feeder abundance was predominantly composed by ophiuroids (Table 2), and their density was positively correlated with xenophyophore test abundance (r_s_ = 0.77–0.79, p < 0.01), as was the density of predator and scavenger fauna (r_s_ = 0.65, p < 0.05). Biological structures can be important in the generation of habitats in the deep sea (Buhl-Mortensen et al., 2010). Such associations are common in the north-eastern Pacific abyss, for instance; sponge stalks can serve as microhabitats for species-rich assemblages of suspension-feeder epifauna (Beaulieu, 2001), or for the attachment of octopod egg clutches during brooding (Purser et al., 2016). Co-occurrence of xenophyophores and ophiuroids has been previously documented in eastern Pacific seamounts (Levin et al., 1986, Levin and Thomas, 1988). Levin (1991) suggested that xenophyophore tests represent a stable substratum that can function as refuge from predators and or nursey habitat for juvenile mobile metazoans, like ophiuroids. Xenophyophore test substratum has been shown to play a crucial role in the regulation of meiofauna and macrofauna communities at the CCZ (Gooday et al., 2017), and our results suggest that these may also be important in the functional structuring of megafauna.

Heterogeneity diversity measures indicated clearly reduced diversity in the Trough relative to Flat and Ridge areas, markedly so in the case of the 1/D index (Fig. 4c). The dominance component of diversity was higher in the Trough (Fig. 5a) unless xenophyophores were included (Fig. 5b). The lower metazoan heterogeneity diversity of the Trough resulted from higher relative abundance of the sponge Porifera msp-5, a taxon possibly better adapted to a presumably more disturbed environmental regime in this area. Porifera msp-5 was amongst the smallest morphospecies we detected (mean diameter 13 mm) and was predominantly found (>70%) encrusting nodules. A recent study revealed a similar dominance, also exhibited by a small nodule-encrusting sponge (*Plenaster craigi*) in the eastern CCZ (Lim et al., 2017). Our results highlight the importance of the standardized detection of small, abundant taxa for robust and comparable assessments of heterogeneity diversity in CCZ megafauna communities.

Previous CCZ megafauna studies have related the presence of nodules with increased metazoan richness (Amon et al., 2016, Tilot et al., 2018, Vanreusel et al., 2016). Although we found no direct correlation between nodule availability and sample diversity, it is possible that the overall lower nodule availability of the Trough played an important role in the reduction of evenness we observed there, since most of the APEI6 metazoan abundance was composed by nodule-dwelling taxa. However, the survey design applied in this study was optimised for the detection of patterns at a relatively broad scale (few kilometres), compared to the tens of meters at which nodule coverage variations usually occur at the CCZ (Peukert et al., 2018). Moreover, our sampling effort evaluation highlighted that two samples did not contain a sufficiently large specimen coverage (<500 ind) to reliably assess richness patterns, and that this may also have affected the estimation of richness in previous studies. Further analysis of our APEI6 dataset may reveal more of the relationships between nodules and megafaunal diversity.

Statistically significant differences in megafaunal density, functional composition, evenness and taxon composition were variously apparent between the landscape types studied. Previous studies have shown that even modest topographic elevation can have substantive effect on abyssal faunal compositions (Durden et al., 2015, Leitner et al., 2017, Stefanoudis et al., 2016). However, in the present study the assemblages of the Flat and Ridge showed a higher similarity, as compared to the Trough area, where most taxon densities were somewhat reduced and the dominant morphospecies shifted from colonial bamboo corals to a small-encrusting sponge. The greater presence of nodule and xenophyophore-test substrata in the Ridge and the Flat possibly increased the environmental heterogeneity of these areas, enhancing the development of more even assemblages. Variations in heterogeneity commonly regulate niche diversification processes (Tews et al., 2004), exerting a fundamental influence on the diversity and structure of deep-sea benthic communities (Levin et al., 2001). Thus, our results suggest that by regulating nodule and xenophyophore test occurrence, and presumably bottom current speeds, geomorphological variations play a crucial role in the structuring of the CCZ megabenthos at the landscape scale.

### 4.4 Ecological significance of megafaunal xenophyophores

Xenophyophore test densities were almost four times higher in Ridge than in the Trough, and almost twice as dense as the Flat. Previous studies have also described higher xenophyophore densities in sites with sloping topography and enhanced water motion (Levin and Thomas, 1988, Stefanoudis et al., 2016). The feeding modes and strategies of xenophyophores remain uncertain (Gooday et al., 1993, Laureillard et al., 2004), with passive particle-trapping, suspension or deposit feeding mechanisms noted (Kamenskaya et al., 2013, Levin and Gooday, 1992). Accepting our inability to distinguish living specimens, that *A. monile* specimens alone represent over 70% of all megafauna observed in the Ridge area suggests considerable ecological significance for this taxon, and the xenophyophores as a group. Note that our identification of 23 xenophyophore morphospecies is undoubtedly an underestimate of their true species diversity (Gooday et al., 2017, Kamenskaya et al., 2013).

Inclusion of xenophyophores substantially affected the assessment of biological diversity, particularly in respect to heterogeneity diversity. It is conceivable that this was a body size mismatch effect. For example, Levin and Gooday (1992) suggest a protoplasm volume of 1–0.01% of test volume. This means that the mean test biomass of *A. monile* at the APEI6 was possibly < 1 mg fwwt ind^−1^ (Gooday et al., 2018), while the mean biomass of the smallest taxa recorded in the metazoan fraction were between 40 and 60 mg fwwt ind^-1^. This mismatch in body sizes suggests that the general interpretation of diversity is probably best limited to the metazoan only assessments.

## 5 Conclusions

This paper presents an assessment of megabenthic faunal distribution in response to seafloor geomorphology at the CCZ. Differences in the megafaunal ecology between landscape types of the APEI6 manifested as changes in standing stock, functional structure, diversity, and community composition. This suggests that the heterogeneity of the abyssal plain habitat can play an important role in the structuring of the CCZ megabenthos, as has been noted with abyssal hills in the NE Atlantic (Durden et al., 2015), and with fish populations in the CCZ (Leitner et al., 2017). We have added a consideration of the trough landscape, where megafauna showed the greatest variations. While regional CCZ benthic ecology has been suggested to be controlled by a gradient of POC flux to the seafloor (Smith et al., 2008b, Veillette et al., 2007), local environmental factors presumably regulated by geomorphology, such as bottom water flows (Mewes et al., 2014), nodule occurrence (Peukert et al., 2018), and xenophyophore test density may be important at the local scale. However, this study lacks replicates of the landscape types studied, and nodule cover variations are assessed at a larger spatial scale than that of their usual variation. Hence, further sampling (in other CCZ ridges, flats, and troughs) along with finer-scale assessments of the influence of nodule resource availability will be required to best interpret the processes leading to the results obtained here. Horst and graben structures (flats, ridges, troughs), and their potential ecological influence, shape most areas of the CCZ seafloor (Macdonald et al., 1996), especially in the centre of this basin (Klitgord and Mammerickx, 1982) where exploration contract areas are located. This complexity needs to be reflected in both local (claim-scale) and regional (CCZ-scale) management plans (Durden et al., 2017a, Levin et al., 2016) and in the design of future monitoring strategies that aim to characterise and preserve biodiversity in the CCZ, as elsewhere in the deep ocean. Our results also indicate the importance of considering sampling unit size in these future assessments.
