# Supplementary material for: Megafaunal variation in the abyssal landscape of the Clarion Clipperton Zone
Source: Prog Oceanogr. 2019 Jan;170:119–33. doi: 10.1016/j.pocean.2018.11.003 (PMC6325340; doi:10.1016/j.pocean.2018.11.003)

**Appendix A**

**Table A.1.** Summary metadata for each sampling unit analysed during the present study. Coordinates (latitude, longitude; in decimal degrees) indicate the central position of each sampling unit. Images (n) are the total number of images processed per transect. Average percentage of polymetallic nodule coverage (± St Dev) was calculated as the mean of the percentage coverages values obtained for each transect image using the CoMoNoD algorithm. Visual annotations: Total abundance of fauna (total counts >10 mm), total morphospecies richness, and percentage of fauna detected on hard substratum (NA), separated into metazoan and xenophyophore taxa. Landscape types: FL= Flat, RI= Ridge, TR=Trough

| Sampling unit | Centre latitude (°) | Centre longitude (°) | Images (n) | Seafloor area (m^2^) | Nodule cover (%) | **Metazoa** | | | **Xenophyophores** | | |
| --- | --- | --- | --- | --- | --- | --- | --- | --- | --- | --- | --- |
|  |  |  |  |  |  | Abundance | Taxa | NA (%) | Abundance | Taxa | NA (%) |
| FL 3 | 17.262 | -123.072 | 774 | 1322 | 12.3 (± 3.2) | 745 | 68 | 75 | 1722 | 18 | 49 |
| FL 33 | 17.233 | -123.027 | 775 | 1321 | 5.4 (± 1.2) | 532 | 76 | 72 | 2952 | 18 | 58 |
| FL 26 | 17.225 | -123.013 | 781 | 1323 | 10.5 (± 4.0) | 634 | 71 | 72 | 2749 | 18 | 55 |
| FL 39 | 17.217 | -123.001 | 778 | 1321 | 12.4 (± 5.6) | 677 | 67 | 56 | 4365 | 19 | 59 |
| RI 2 | 17.282 | -122.878 | 720 | 1324 | 9.1 (± 8.0) | 537 | 63 | 65 | 5687 | 20 | 36 |
| RI 9 | 17.297 | -122.883 | 729 | 1322 | 5.5 (± 2.1) | 552 | 67 | 61 | 4731 | 20 | 28 |
| RI 15 | 17.310 | -122.888 | 666 | 1323 | 3.6 (± 1.1) | 629 | 59 | 47 | 4508 | 19 | 39 |
| RI 21 | 17.323 | -122.891 | 765 | 1321 | 6.2 (± 7.5) | 720 | 70 | 69 | 6379 | 20 | 30 |
| TR 15 | 17.264 | -122.830 | 694 | 1324 | 1.7 (± 1.4) | 382 | 54 | 60 | 1202 | 18 | 26 |
| TR 18 | 17.248 | -122.821 | 555 | 1322 | 8.0 (± 3.4) | 506 | 72 | 43 | 4217 | 18 | 52 |
| TR 25 | 17.223 | -122.817 | 709 | 1324 | 3.2 (± 2.3) | 546 | 65 | 67 | 1237 | 19 | 45 |
| TR 29 | 17.220 | -122.820 | 623 | 1323 | 1.8 (± 1.9) | 280 | 47 | 56 | 419 | 18 | 37 |

**Fig A.1.** Sediment grain-size distributions plots generated for different sediment horizons sampled at the APEI6 seafloor. Lines representing mean frequency across each of the five replicate megacore samples collected per landscape type. Shadowed areas representing maximum and minimum values per replicate set. Each core was initially sliced and split into nine different sediment depths (0-5, 5-10, 10-15, 15-20, 20-30, 30-50, 50-100, 100-150, and 150-200 mm). Sediment grain-size distributions at each horizon were measured independently by laser diffraction. Horizons 0-5, 5-10, 10-15, 15-20, 20-30, 30-50 were averaged into a 0-50 mm depth.


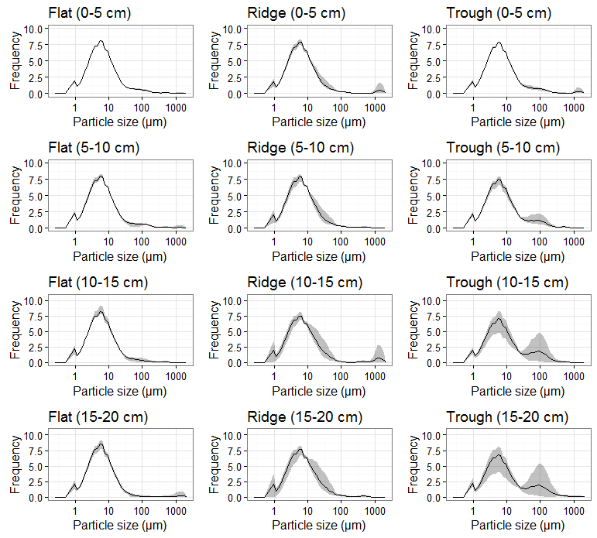


**Table A.2.** Particle size statistics calculated applying a geometric method of moments for different sediment horizons sampled at the APEI6 seafloor. Values representing maximum and minimum ranges across each of the five replicate megacore samples collected per landscape type. Each core was initially sliced and split into nine different sediment depths (0-5, 5-10, 10-15, 15-20, 20-30, 30-50, 50-100, 100-150, and 150-200 mm). Sediment grain-size distributions at each horizon were measured independently by laser diffraction. Horizons 0-5, 5-10, 10-15, 15-20, 20-30, 30-50 were averaged into a 0-50 mm depth, prior to the statistical processing.

| **Horizon** | **Statistic** | **Flat** | **Ridge** | **Trough** |
| --- | --- | --- | --- | --- |
| 0 to 5 cm | Mean | 7.15 - 7.61 | 6.71 - 9.21 | 7.60 - 8.50 |
|  | St dev | 2.82 - 3.03 | 2.54 - 4.77 | 2.99 - 4.04 |
|  | Skewness | 0.96 - 1.50 | 0.46 - 2.02 | 0.86 - 1.86 |
|  | Kurtosis | 4.50 - 7.35 | 3.22 - 8.29 | 3.79 - 7.50 |
|  | Mode | 7.19 | 7.19 | 7.19 |
|  | D_50_ | 6.47 - 6.70 | 6.29 - 7.40 | 6.61 - 7.03 |
| 5 to 10 cm | Mean | 6.50 - 8.52 | 6.56 - 8.72 | 7.49 - 11.16 |
|  | St dev | 2.73 - 3.66 | 2.71 - 2.78 | 2.95 - 3.97 |
|  | Skewness | 0.89 - 1.56 | 0.46 - 1.15 | 0.67 - 1.07 |
|  | Kurtosis | 4.18 - 6.60 | 3.20 - 5.82 | 2.73 - 5.20 |
|  | Mode | 7.19 | 7.19 | 7.19 |
|  | D_50_ | 5.97 - 6.89 | 5.98 - 7.95 | 6.63 - 8.17 |
| 10 to 15 cm | Mean | 6.06 - 7.24 | 6.33 - 11.67 | 6.47 - 20.08 |
|  | St dev | 2.10 - 3.00 | 2.43 - 6.73 | 2.48 - 4.72 |
|  | Skewness | 0.06 - 1.08 | 0.12 - 1.64 | 0.06 - 0.90 |
|  | Kurtosis | 2.75 - 5.02 | 2.34 - 6.26 | 1.79 - 6.14 |
|  | Mode | 7.19 | 7.19 | 7.19 |
|  | D_50_ | 6.04 - 6.48 | 5.87 - 9.50 | 6.29 - 16.45 |
| 15 to 20 cm | Mean | 5.77 - 8.55 | 6.07 - 10.61 | 6.35 - 20.15 |
|  | St dev | 2.19 - 4.28 | 2.50 - 2.94 | 2.56 - 5.07 |
|  | Skewness | 0.01 - 1.93 | 0.13 - 1.18 | - 0.14 - 1.85 |
|  | Kurtosis | 2.59 - 7.50 | 2.35 - 6.17 | 1.77 - 8.79 |
|  | Mode | 7.19 | 7.19 | 7.19 - 115.00 |
|  | D_50_ | 5.69 - 6.63 | 5.70 - 10.28 | 5.93 - 31.85 |

**Fig. A.2**. Morphospecies rarefaction curves extrapolated to ~three times the area sampled at each landscape type for the present study. Triangles showing the total size of the sample analysed at each geomorphology. Expected richness with sample coverages of 15,000 m^2^ show a lower richness at the Trough (~107 msp) compared to the Flat (~130 msp) and the Ridge (~134 msp) areas, but confidence intervals continued to overlap between curves. Whole geomorphological units sample size (5280 m^2^) covered 85-90% of the expected richness > 15,000 m^2^.


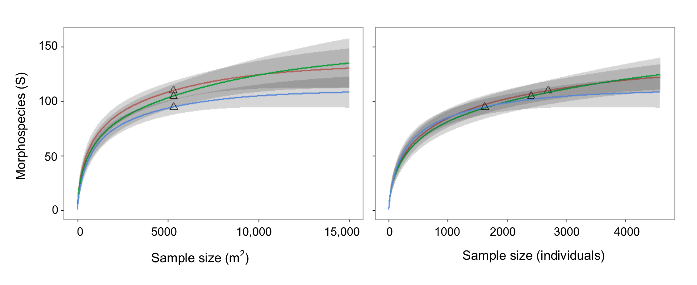


**Fig. A.3.** Metazoan morphospecies surveyed for the present study. Venn diagram showing the total number of metazoan taxa shared between each combination of landscape types of the APEI6. *In brackets*: singleton morphospecies.


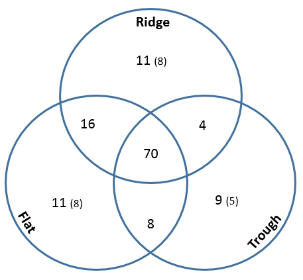


**Fig. A.4.** Variations of the coefficient of variation with increasing sample size calculated for the main ecological estimators used in the present study. Coefficients of variation were calculated as the standard deviation divided by the mean of each estimator at each different sampling effort (see methods), for the whole metazoan dataset collected for each landscape type of the APEI6.


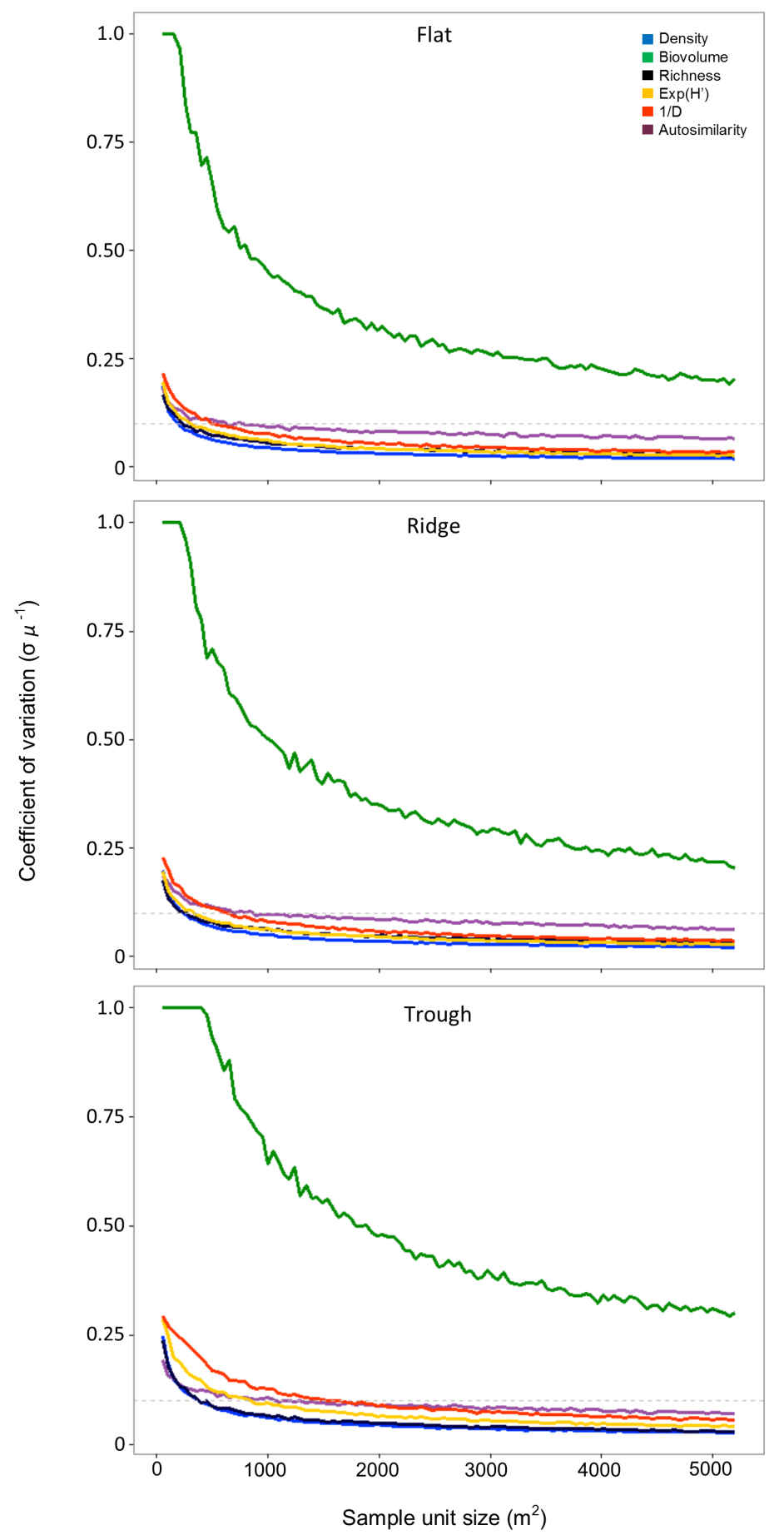


**Fig. A.5.** Relative variations of the coefficient of variation with increasing sample size calculated for the main ecological estimators used in the present study. Coefficients of variation were calculated as the standard deviation divided by the mean of each estimator at each different sampling effort (see methods), for the whole metazoan dataset collected for each landscape type of the APEI6, and then divided by the minimum value exhibited in each along the sample size spectrum assessed.


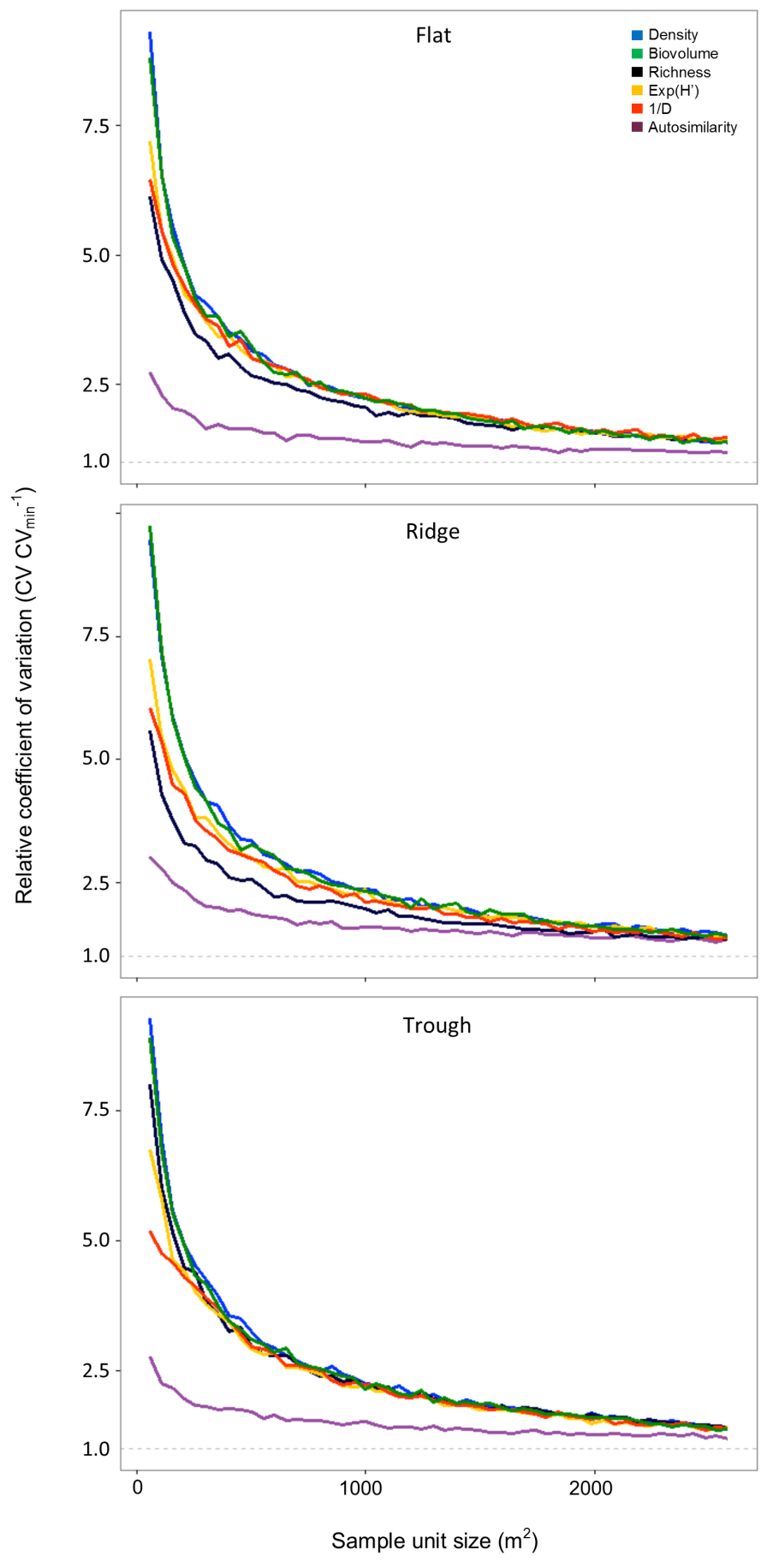

Supplement: Supplementary data 1 [file mmc1.docx]
